# Social Stratification and Tooth Loss Among Migrant and Non-Migrant Middle-Aged and Older Adults in China

**DOI:** 10.1093/geroni/igaa057.3050

**Published:** 2020-12-16

**Authors:** Xiaomin Qu, Bei Wu, Jiaojiao Yu, Haidong Zhang

**Affiliations:** 1 East China University of Political Science and Law, Shanghai, China; 2 New York University, New York, New York, United States; 3 School of History and Ethnology, Qiannan Normal University for Nationalities, Duyun, Guizhou, China; 4 School of Sociology and Political Science, Shanghai University, Shanghai, China

## Abstract

This study investigated the association between socioeconomic status (SES) and tooth loss in middle-aged and older adults by migrant status. The sample included 2,390 participants aged 45-65 from the 2017 Urbanization and New Migrant Survey conducted from 10 cities in China. Results from the negative binomial regression and the marginal effect analysis showed that education, income, and residence in a developed city were negatively associated with tooth loss for non-migrants and migrants with high levels of education. These associations were not found to be significant for migrants with low education levels. The findings suggest that SES plays a more significant role in tooth retention for migrants with higher education levels compared to those with lower education levels. These results may largely be due to different levels of health literacy and unequal access to dental care services. Tailored intervention needs to be target migrant populations with low SES.

